# Sexual and reproductive health and rights of migrants: strengthening regional research capacity

**DOI:** 10.2471/BLT.20.270447

**Published:** 2021-03-10

**Authors:** Vanessa Brizuela, Anuj Kapilashrami, Mercedes Bonet, Rajat Khosla, Loulou Kobeissi, Lale Say, Anna Thorson

**Affiliations:** a UNDP/UNFPA/UNICEF/WHO/World Bank Special Programme of Research, Development and Research Training in Human Reproduction (HRP), Department of Sexual and Reproductive Health and Research, World Health Organization, 20 Avenue Appia, 1211 Geneva 27, Switzerland.; b School of Health and Social Care, University of Essex, Essex, England.

In the past few years, the numbers of refugees and migrants have increased worldwide, with a reported 79.5 million people forcibly displaced in 2019, the highest number ever recorded.

Migrants and refugees are exposed to complex social, ethnic-based, political and economic hardships that undermine their fundamental right to health and predispose them to immediate disparities in morbidity and mortality. Research highlights the stigma, discrimination and barriers that migrants face to access care.^1^^,^^2^ As with many public health emergencies, the coronavirus disease 2019 pandemic has exposed the plight of migrants; they are frequently neglected in national health policies, and health systems struggle to respond promptly and effectively to their health needs.^3^ Because of their status, migrants often have their sexual and reproductive health and rights curtailed, if not totally disregarded by their governments, host governments and the international community.^4^ This gap includes access to maternity services, comprehensive abortion care, contraceptive services, management and prevention of sexually transmitted infections including human immunodeficiency virus, and responding to the needs of survivors of gender-based violence, among others.^5^ In Colombia, for example, recent reports from Venezuelan migrants provide evidence of increased gender-based violence, unmet family planning needs, and inadequate access to maternal health-care services.^6^

The 2017 World Health Assembly Resolution WHA70.15 on the promotion of the health of refugees and migrants recognizes the issues migrants face: the resolution brought about a draft global action plan identifying the priority areas of work. Similarly, the UCL-*Lancet* Commission on Migration and Health highlighted the need to provide high quality care to migrants, especially within the context of the sustainable development goals and achieving universal health coverage.^4^

To best respond to the various challenges and sexual and reproductive health and rights needs of migrants and refugees, we need high-quality and context-specific research based on local and regional priorities.^7^ However, to facilitate generation of local evidence, a critical – but unmet – aspect is strengthening research capacity, to ensure that local investigators can lead the production and dissemination of knowledge that most closely relates to them and their communities.^8^^,^^9^

The United Nations Development Programme/United Nations Population Fund/United Nations Children’s Fund/World Health Organization/World Bank Special Programme of Research, Development and Research Training in Human Reproduction has been leading research and developing research capacity in sexual and reproductive health and rights for several decades,^10^ through its large network of local research partners and currently through the programme’s Reproductive Health Programme Alliance for Research Capacity Strengthening.^11^ The programme has extensively worked in humanitarian settings, such as in Bangladesh, Democratic Republic of the Congo, and Yemen, assessing barriers in access to sexual and reproductive health and rights services.^12^ The programme supported a research prioritization exercise in collaboration with the Inter-Agency Working Group on Reproductive Health in Crises to ensure the inclusion of cross-cutting themes around universal access to quality and comprehensive sexual and reproductive health and rights services. Furthermore, the alliance has been supporting locally led research in emergency situations and fostering research capacity strengthening through small grant schemes responding to disease outbreaks or humanitarian crises.^8^

Here, we draw on experiences from the alliance in research capacity strengthening efforts during emergencies to identify key lessons as well as principles to guide research capacity strengthening activities that respond to global health needs as well as influence health agendas. Decades of work in research capacity strengthening have shown that such initiatives must consider and address gender and social disparities in the distribution of research capacities and resources. The programme’s Gender and Rights Advisory Panel has been critical in clarifying these aspects and ensuring our research initiatives are mindful and intentional in integrating considerations for gender and human rights. Adopting a gender and rights lens is particularly relevant, considering that migrant and refugee voices have been frequently unheard, and women and researchers located in low- and middle-income countries underrepresented. Research capacity strengthening initiatives demand special attention to ethical and methodological challenges of studying migrants’ lives and health for two important reasons. First, the population of interest are usually stigmatized regardless of their legal status and often afraid of seeking care through the formal health systems. Second, their participation in research may carry risks of identification that could lead to additional marginalization and/or deportation in certain contexts. The research methods and data collection tools used, as well as the informed consent process during the conduct of research with migrants, need to be given special attention to ensure that the voice, perceptions and experiences of migrants are well represented.^4^ Such efforts include sensitizing researchers on the range of participatory methods available to meaningfully engage migrants and refugees in studies, and spearheading of research initiatives by local partners to ensure that findings are translated into action and inform policy. It is also necessary to strengthen research ethics committees so that they can best respond to studies aimed at working with migrants and refugees. Skills and capacities strengthening must be simultaneously driven by extending opportunities for funding and research collaboration to researchers in each region.

In 2019, the alliance issued a call for proposals to support local research teams looking at the consequences of the mass migration movement in the World Health Organization Region of the Americas on sexual and reproductive health and rights. This call was a collaboration with the Special Programme for Research and Training in Tropical Diseases, the Alliance for Health Policy and Systems Research, and the Pan American Health Organization (PAHO), in response to raising concerns over the challenges faced by Venezuelans and Central Americans as they migrate towards other countries in the region; the call for proposals was based on the globally prioritized sexual and reproductive health and rights questions.

We draw on the experience in engaging in research capacity strengthening, and this call, to reflect upon our learnings from the past and the specific needs presented by the migration crisis in the Region of the Americas. A unique and open process of calling for and screening was adopted to assess over 60 proposals. The process involved the creation of an ad hoc review team that included staff from all sponsoring organizations, as well as the alliance hub for Latin America (*Centro de Pesquisas em Saúde Reprodutiva de Campinas*) in Brazil, and guidance from the programme’s Gender and Rights Advisory Panel, on how gender and other social equities could be embedded throughout the research development and implementation. The goal was to ensure that the proposals were scientifically sound and methodologically feasible, that assurances to gender and human rights were included in the proposals, and that specific foci on research capacity strengthening activities and goals were central to the projects. In an effort to further foster local capacity and capacity strengthening activities, the hub for Latin America was entrusted with both coordinating and managing the call, and also with leading all research capacity strengthening activities relating to the call. These responsibilities included support throughout submission and response to ethics review boards both locally and globally through PAHO’s Ethics Review Committee, and in protocol development and data collection plans, as well as for future assistance in data analysis and manuscript writing. The research projects are ongoing and the research groups are making efforts to overcome the obstacles posed by the pandemic, which affects not only the population of interest but also data collection. The institutions leading the 12 selected projects will continue to collaborate with the hub for Latin America as part of the alliance but also with the programme through research projects aimed at improving sexual and reproductive health and rights in the region, and in particular among migrants.

In an ever-changing global environment, responding to the vulnerabilities of migrants and refugees compels the need for timely data around their sexual and reproductive health and rights needs and health system response in host countries, and for research capacity strengthening. The alliance, through its regional presence, can provide an agile and context-specific solution by supporting local, gender-balanced research teams in evidence generation and giving voice to migrants and refugees by using appropriate methods. The alliance can provide support and mentorship to these groups through institutions located in the region. The goal of said support and mentorship is to strengthen research protocols, ensure considerations for ethics and data collection among migrants and refugees are included, and aid in the analysis of data and publication of results in peer-reviewed journals, making equitable considerations for authorship. The benefits of this approach are both with regards to the advancement of knowledge and to sustainable mechanisms for locally led studies. Improved research capacity strengthening can help build a better health system response that is context specific and addresses the needs of migrants and refugees.

The global health community needs to lead and coordinate efforts in ensuring that sexual and reproductive health and rights of migrants are secured and promoted through the implementation of locally generated and regionally supported, evidence-based policies. Ensuring that local researchers can respond rapidly and effectively to emergencies is an integral component of research capacity strengthening initiatives.
